# Chronic Obstructive Pulmonary Disease Is Associated With Decreased Quality of Life in Bronchiectasis Patients: Findings From the KMBARC Registry

**DOI:** 10.3389/fmed.2021.722124

**Published:** 2021-08-16

**Authors:** Sang Hyuk Kim, Changhwan Kim, Ina Jeong, Seung Jun Lee, Tae Hyung Kim, Chang Youl Lee, Yeon-Mok Oh, Hyun Lee, Youlim Kim

**Affiliations:** ^1^Division of Pulmonology and Critical Care Medicine, Samsung Medical Center, Department of Medicine, Sungkyunkwan University School of Medicine, Seoul, South Korea; ^2^Department of Internal Medicine, Jeju National University Hospital, Jeju National University School of Medicine, Jeju, South Korea; ^3^Department of Internal Medicine, National Medical Center, Seoul, South Korea; ^4^Division of Pulmonology and Allergy, Department of Internal Medicine, Gyeongsang National University Hospital, Gyeongsang National University School of Medicine, Jinju, South Korea; ^5^Division of Pulmonary Medicine and Allergy, Department of Internal Medicine, Hanyang University College of Medicine, Seoul, South Korea; ^6^Division of Pulmonary, Allergy, and Critical Care Medicine, Department of Internal Medicine, Hallym University Chuncheon Sacred Heart Hospital, Chuncheon, South Korea; ^7^Clinical Research Center for Chronic Obstructive Airway Diseases, Asan Medical Center, Department of Pulmonary and Critical Care Medicine, University of Ulsan College of Medicine, Seoul, South Korea

**Keywords:** chronic obstructive pulmonary disease, bronchiectasis, quality of life, bronchiectasis health questionnaire, COPD overlap

## Abstract

Most studies have evaluated the impact of non-cystic fibrosis bronchiectasis (hereafter referred to as bronchiectasis) on quality of life (QoL) in patients with chronic obstructive pulmonary disease (COPD) using COPD cohorts. Accordingly, the impact of COPD on QoL in patients with bronchiectasis is not well-elucidated. We used the Korean Multicenter Bronchiectasis Audit and Research Collaboration (KMBARC) registry between August 2018 and December 2019, a prospective observational cohort that enrolled patients with bronchiectasis in Korea. We evaluated co-occurrence exposure to COPD in bronchiectasis patients, and the primary outcome was QoL according to the Bronchiectasis Health Questionnaire (BHQ). We also investigated factors associated with decreased QoL, defined as the lowest quartile of the total BHQ score. Of 598 patients with bronchiectasis, 372 (62.2%) had COPD. Bronchiectasis patients with COPD had a significantly lower total BHQ score compared with those without COPD [median = 63.1 (interquartile range: 54.8–68.6) vs. 64.8 (57.4–70.8), *p* = 0.020]. Multivariable analysis revealed that dyspnea [adjusted odds ratio (aOR) = 3.21, 95% confidence interval (CI) = 1.21–8.60], depression (aOR = 1.28, 95% CI = 1.16–1.44), and fatigue (aOR = 1.05, 95% CI = 1.01–1.09) were significantly associated with decreased QoL in bronchiectasis patients with COPD. In conclusion, bronchiectasis patients with COPD had significantly decreased QoL than patients without COPD. In bronchiectasis patients with COPD, dyspnea, depression, and fatigue were associated with decreased QoL.

## Introduction

Chronic obstructive pulmonary disease (COPD) is a common pulmonary comorbidity of non-cystic fibrosis bronchiectasis (hereafter referred to as bronchiectasis), ranging from 15 to 38% prevalence in bronchiectasis patients (1–5). These two conditions facilitate each other's disease progression (6), resulting in a worse prognosis, including mortality.

However, except for studies evaluating COPD prevalence in patients with bronchiectasis, most studies have focused on the impact of bronchiectasis in COPD patients (7–10). These studies showed that the coexistence of bronchiectasis and COPD was associated with a larger number of symptoms, worse lung function, greater disease severity, and more frequent exacerbations (7–10). Accordingly, COPD patients with bronchiectasis have worse quality of life (QoL) than patients with COPD only (11, 12). Because these studies used COPD cohorts, the impact of bronchiectasis on QoL in COPD patients could have been elucidated better. However, little is known about this association within a bronchiectasis cohort because few studies have evaluated bronchiectasis cohorts; thus, urgent evaluation of this issue is necessary.

The Korean Multicenter Bronchiectasis Audit and Research Collaboration (KMBARC) is a prospective, observational study of bronchiectasis in Korea (13, 14). The KMBARC uses the Korean version of the Bronchiectasis Health Questionnaire (BHQ) for QoL measurement (15), which has the advantage of simplicity over other QoL measurements for bronchiectasis. Accordingly, we hypothesized that the BHQ QoL would be worse in bronchiectasis patients with COPD than in those without COPD. We further aimed to evaluate which factors are associated with impaired QoL in bronchiectasis patients with COPD.

## Materials and Methods

### Study Population

The KMBARC is a prospective, non-interventional observational cohort study conducted since August 2018 to uncover the natural course of bronchiectasis in Korea. We used baseline data from 598 participants enrolled between August 2018 and December 2019. The KMBARC inclusion criteria were adult patients (aged ≥ 18 years) with stable bronchiectasis. In this study, bronchiectasis was defined when bronchodilation was found in computed tomography of the lung, which included one or more of the followings: (1) bronchoarterial ratio > 1 (internal airway lumen vs. adjacent pulmonary artery), (2) lack of tapering, or (3) airway visibility within 1 cm of costal pleural surface or touching mediastinal pleura (16). The exclusion criteria were (1) cystic fibrosis bronchiectasis, (2) interstitial lung-disease-related traction bronchiectasis, (3) active treatment for pneumonia, pulmonary tuberculosis (TB), or non-tuberculous mycobacterial infection, (4) lack of informed consent, and (5) pregnancy. Detailed information on the KMBARC protocol was provided in a previous study (13).

The study protocol was approved by the institutional review board of each institution that participated in the KMBARC, including Hallym University Chuncheon Sacred Heart Hospital (IRB number: 2018-07-006). All participants provided written informed consent.

### Exposure

The exposure state for this study was the coexistence of COPD with bronchiectasis. COPD was defined as physician-diagnosed COPD, which includes (1) COPD diagnosis by an attending physician in the institution where the patient was enrolled and (2) Patient-reported physician-diagnosed COPD in another institution.

### Outcomes

The primary outcome was QoL as measured by BHQ (17). The secondary outcome was factors associated with decreased QoL, defined as the lowest quartile of the total BHQ score.

### Covariates

Body mass index (BMI) was calculated by dividing weight by the square of height (kg/m^2^). Dyspnea was evaluated according to the modified Medical Round Council (mMRC) scale (18). Purulent sputum production was assessed using a sputum color chart (19). Acute exacerbation was defined according to a consensus definition for bronchiectasis (20). Regarding acute exacerbations, we evaluated exacerbation history in the previous year at the time of enrollment. Bronchiectasis severity was assessed by the bronchiectasis severity index (BSI) (21) and FACED score (22). *Pseudomonas aeruginosa* was isolated from spontaneously obtained sputum, induced sputum samples, or bronchoalveolar lavage. Korean versions of the Patient Health Questionnaire 9 (PHQ-9) and the Fatigue Severity Score (FSS) were used to assess depression and fatigue, respectively (23–26). Modified Reiff score was calculated as previously reported (27). Comorbidities were defined as patient-reported previous physician diagnoses. Spirometry was performed as recommended by the American Thoracic Society and the European Respiratory Society (28). After obtaining absolute values for FEV_1_ and FVC, the percentages of predicted values (% predicted) for FEV_1_ and FVC were calculated following recommendations for Korean populations (29). Medication data were based on self-reported use.

### Statistical Analyses

We presented data as medians with interquartile ranges (IQRs) for continuous variables and numbers with percentages for categorical variables. The *p*-values were calculated using the Wilcoxon rank-sum test for continuous variables and Pearson's chi-square test or Fisher's exact test for categorical variables, as appropriate. We performed univariable and multivariable logistic regression analyses to identify factors associated with decreased QoL in bronchiectasis patients with COPD. Factors included in the multivariable logistic regression model were demographics (age, sex, and smoking history), clinically important variables (acute exacerbation), and factors significantly different (*p* < 0.05) between the bronchiectasis patients with and without COPD (BMI, mMRC, PHQ-9, FSS, FEV_1_ % predicted, asthma, modified Reiff score). As each component of BSI or FACED was included in the model, these variables were not included in the multivariable models. In addition, due to the high collinearity between pulmonary function parameters, only FEV_1_ % predicted was adjusted. A two-sided *p*-value < 0.05 was considered significant. All analyses were conducted using R version 4.0.3 (R Core Team 2020; R Foundation for Statistical Computing, Vienna, Austria).

## Results

### Baseline Characteristics

Of 598 bronchiectasis patients, 226 (37.8%) had COPD (Table 1). Compared with patients without COPD, bronchiectasis patients with COPD were older [median 67 years (IQR, 60–72 years) vs. median 65 years (IQR, 60–71 years), *p* = 0.025], more frequently male (59.3 vs. 34.9%, *p* < 0.001), and more likely to be current or ex-smokers (49.6 vs. 26.6%, *p* < 0.001). Regarding symptoms, compared with patients without COPD, while dyspnea (mMRC ≥ 2) (31.9 vs. 15.6%, *p* < 0.001) was more common, purulent sputum production (20.7 vs. 34.2%, *p* < 0.001) was less frequent in patients with COPD. Regarding disease severity, bronchiectasis patients with COPD had higher BSI [median 7 (IQR, 5–11) vs. median 5 (IQR, 4–8), *p* < 0.001] and FACED [median 3 (IQR, 1–4) vs. median 1 (IQR, 0–3), *p* < 0.001] than patients without COPD. Additionally, *Pseudomonas aeruginosa* was more commonly isolated from bronchiectasis patients with COPD than patients without COPD (14.6 vs. 8.9%, *p* = 0.042). However, there was no intergroup difference in acute exacerbation. Patients with COPD had a higher number of involved lobes than those without COPD [median 4 (IQR, 2–5) vs. median 3 (IQR, 2–4), *p* < 0.001]. In addition, cystic bronchiectasis was more frequently found in patients with COPD than those without COPD (56 vs. 40.9%, *p* = 0.001). Regarding comorbidities, asthma (28.8 vs. 18.5%, *p* = 0.005), cardiovascular disease (38.1 vs. 24.7%, *p* < 0.001), and tuberculosis (42.7 vs. 27.4%, *p* < 0.001) were more common in bronchiectasis patients with COPD than in patients without bronchiectasis. Bronchiectasis patients with COPD showed lower pulmonary function in terms of FVC (% predicted), FEV_1_ (L), FEV_1_ (% predicted), and FEV_1_/FVC than those without COPD (*p* < 0.001 for all). Bronchiectasis patients with COPD were more frequently prescribed a long-acting muscarinic antagonist (LAMA) or a long-acting β_2_ agonist (LABA) (*p* = 0.12), LABA/LAMA (*p* < 0.001), or an inhaled corticosteroid (ICS)/LABA/LAMA (*p* < 0.001) than were patients without COPD. However, there were no differences in use of ICS/LABA (*p* = 0.866), statins (*p* = 0.592), angiotensin-converting enzyme inhibitors (*p* = 0.189), or proton pump inhibitors (*p* = 0.906) between the two groups.

**Table 1:** Clinical characteristics of the study population according to COPD status.

	**Without COPD (*n* = 372)**	**With COPD (*n* = 226)**	***p*-value**
Age, years	65 (60–71)	67 (60–72)	0.025
Male, *n* (%)	130 (34.9)	134 (59.3)	<0.001
BMI, kg/m^2^ (*n* = 562)	23 (21–25)	23 (21–26)	0.283
Current or ex-smoker, *n* (%)	99 (26.6)	112 (49.6)	<0.001
**Symptoms**			
mMRC ≥ 2, *n* (%)	58 (15.6)	72 (31.9)	<0.001
Purulent sputum production (*n* = 583)	125 (34.2)	45 (20.7)	<0.001
**Disease severity**			
Acute exacerbation, *n* (%)	189 (50.8)	133 (58.8)	0.067
Severity index (*n* = 582)			
BSI	5 (4–8)	7 (5–11)	<0.001
FACED	1 (0–3)	3 (1–4)	<0.001
Isolation of *Pseudomonas aeruginosa, n* (%)	33 (8.9)	33 (14.6)	0.042
**Health status**			
PHQ-9	3 (1–9)	3 (1–9)	0.909
FFS	20 (12–34)	20 (12–34)	0.990
Radiologic findings (*n* = 582)			
Number of involved lobes	3 (2–4)	4 (2–5)	<0.001
Cystic bronchiectasis in any lobes	149 (40.9)	122 (56.0)	0.001
Modified Reiff score (*n* = 569)	4 (3–7.5)	6 (3–10)	<0.001
**Comorbidities**			
Asthma, *n* (%)	69 (18.5)	65 (28.8)	0.005
Cardiovascular disease, *n* (%)	92 (24.7)	86 (38.1)	<0.001
Diabetes mellitus, *n* (%) (*n* = 597)	48 (12.9)	25 (11.1)	0.604
Rhinosinusitis, *n* (%)	33 (8.9)	20 (8.8)	1.000
Neoplastic disease, *n* (%) (*n* = 594)	31 (8.4)	23 (10.2)	0.547
Tuberculosis, *n* (%) (*n* = 597)	102 (27.4)	96 (42.7)	<0.001
**Pre-bronchodilator spirometry results**			
FVC (L)	2.5 (2.0–3.1)	2.4 (1.9–3.0)	0.104
FVC (%-predicted)	75.7 (66.7–86.7)	67.9 (55.8–79.0)	<0.001
FEV_1_ (L)	1.8 (1.4–2.1)	1.4 (1.0–1.8)	<0.001
FEV_1_ (%-predicted)	71.2 (57.3–82.4)	53.2 (42.3–64.2)	<0.001
FEV_1_/FVC (%)	71.6 (63.2–77.1)	59.2 (50.3–65.8)	<0.001
**Medication**			
Inhaler (*n* = 595)			
LABA or LAMA, *n* (%)	17 (4.6)	23 (10.3)	0.012
ICS/LABA^*^, *n* (%)	36 (9.7)	20 (8.9)	0.866
LABA/LAMA^*^, *n* (%)	49 (13.2)	107 (47.8)	<0.001
ICS/LABA/LAMA, *n* (%)	16 (4.3)	39 (17.4)	<0.001
Oral drug (*n* = 596)			
Statin, *n* (%)	21 (5.7)	16 (7.1)	0.592
ACE inhibitor, *n* (%)	8 (2.2)	1 (0.4)	0.189
Proton pump inhibitor, *n* (%)	29 (7.8)	19 (8.4)	0.906

*Data are expressed as medians (interquartile ranges) for continuous variables and numbers (percentages) for categorical variables*.

* *Seven patients switched from one inhaler to the other*.

*COPD, chronic obstructive pulmonary disease; BMI, body mass index; mMRC, modified Medical Research Council; FEV_1_, forced expiratory volume in 1 second; FVC, forced vital capacity; BSI, bronchiectasis severity index; PHQ-9, Patient Health Questionnaire 9; FSS, Fatigue Severity Score; LABA, long-acting β_2_ agonist; LAMA, long-acting muscarine antagonist; ICS, inhaled corticosteroid; ACE, angiotensin-converting enzyme*.

### Comparison of QoL Between Bronchiectasis Patients With and Without COPD

**Figure 1:**
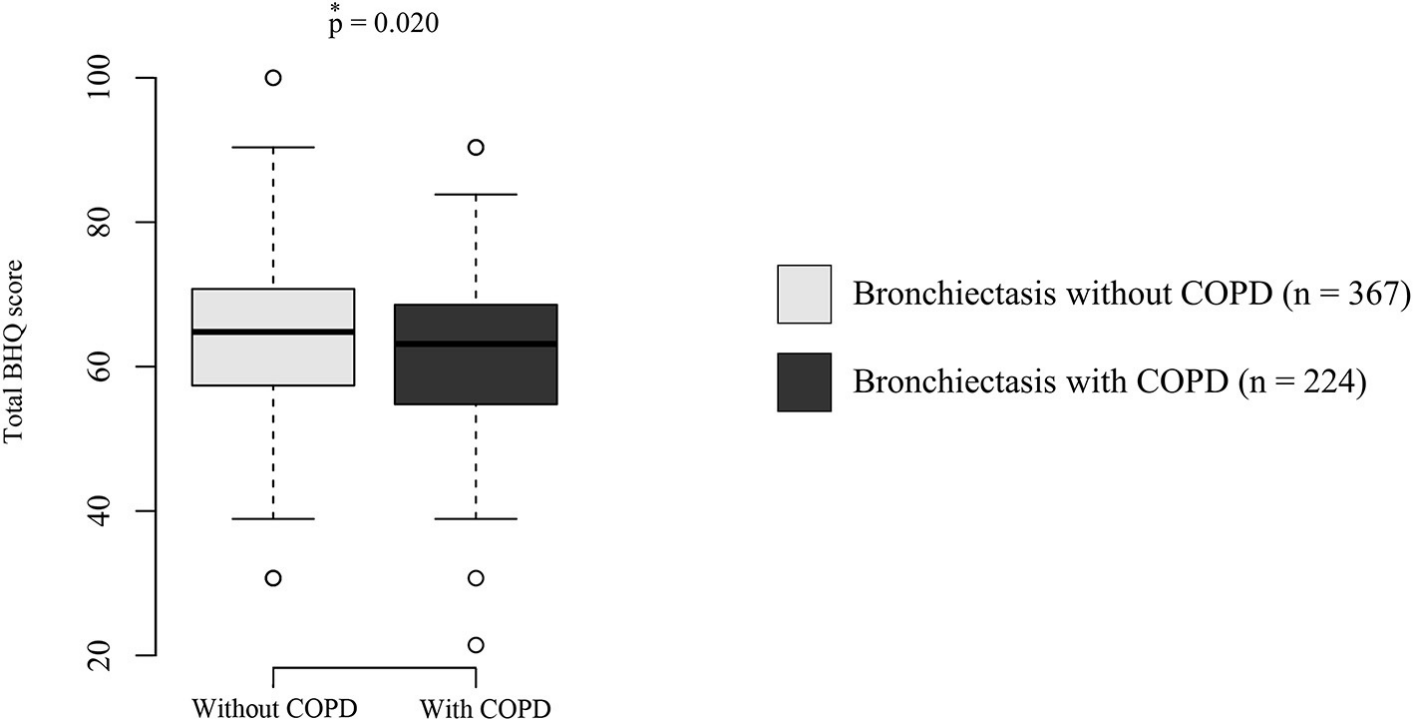
Quality of life in bronchiectasis patients with or without COPD. The boxplot indicates maximum, first quartile, median, third quartile, and minimum QoL scores. Dots outside of boxplots indicate outliers. The *p*-value was calculated using the Wilcoxon rank-sum test. COPD, chronic obstructive pulmonary disease; BHQ, bronchiectasis health questionnaires.

Figure 1 depicts the comparison of the total BHQ score according to the presence of COPD with bronchiectasis. Bronchiectasis patients with COPD had significantly lower total BHQ scores than patients without COPD [median 63.1 (IQR, 54.8–68.6) vs. median 64.8 (IQR, 57.4–70.8), *p* = 0.020].

### Comparison of Characteristics of Bronchiectasis Patients With COPD According to QoL

As shown in Table 2, there were no significant differences in age (*p* = 0.688), sex (*p* = 0.530), smoking history (*p* = 0.425), purulent sputum production (*p* = 0.657), acute exacerbation (*p* = 0.209), the isolation of *pseudomonas aeruginosa* (*p* = 0.238), comorbidities, and medications between the bronchiectasis patients with COPD showing decreased QoL and those not. However, the proportion of patients with mMRC ≥ 2 was significantly higher in patients with decreased QoL than in those without decreased QoL (63.1 vs. 18.9%, *p* < 0.001). BMI [median 21.7 kg/m^2^ (IQR, 20.1–24.6 kg/m^2^) vs. median 23.6 kg/m^2^ (IQR, 21.0–26.0 kg/m^2^), *p* = 0.007] and pulmonary function, including FVC % predicted [median 62.3 % (IQR, 50.3–75.8%) vs. median 69.7% (IQR, 59.0–79.2%), *p* = 0.003] and FEV_1_ % predicted [median 46.9% (IQR, 35.1–59.6%) vs. median 60.3% (IQR, 51.9–65.7%), *p* = 0.002] were significantly lower in patients with decreased QoL compared to those without decreased QoL. In contrast, BSI [median 8.5 (IQR, 6–14) vs. median 6 (IQR, 5–9), *p* < 0.001], FACED [median 37 (IQR, 28–51) vs. median 15 (IQR, 11–23), *p* < 0.001], PHQ-9 [median 11 (IQR, 7–17) vs. median 2 (IQR, 1–4), *p* < 0.001], and FFS [median 37 (IQR, 28–51) vs. median 15 (IQR, 11–23), *p* < 0.001] were significantly higher in patients with decreased QoL than those without decreased QoL.

**Table 2:** Baseline characteristics of bronchiectasis patients with COPD according to QoL.

	**Bronchiectasis patients with COPD (** ***N*** **= 224)**		***p*-value**
	**(** ***N*** **= 224)**		
	**Without decreased QoL**	With decreased QoL^*^	
	**(*n* = 159)**	**(*n* = 65)**	
Age, years	66 (61–72)	68 (60–72)	0.688
Male, *n* (%)	97 (61.0)	36 (55.4)	0.530
BMI, kg/m^2^ (*n* = 221)	23.6 (21.0–26.0)	21.7 (20.1–24.6)	0.007
Current or ex-smoker, *n* (%)	82 (51.6)	29 (44.6)	0.425
**Symptoms**			
mMRC ≥ 2, *n* (%)	30 (18.9)	41 (63.1)	<0.001
Purulent sputum production (*n* = 215)	33 (21.6)	11 (17.7)	0.657
**Disease severity**			
Acute exacerbation, *n* (%)	89 (56.0)	43 (66.2)	0.209
Severity index (*n* = 216)			
BSI	6 (5–9)	8.5 (6–14)	<0.001
FACED	15 (11–23)	37 (28–51)	<0.001
Isolation of *Pseudomonas aeruginosa, n* (%)	21 (13.2)	12 (18.5)	0.238
**Health status**			
PHQ-9	2 (1–4)	11 (7–17)	<0.001
FFS (*n* = 223)	15 (11–23)	37 (28–51)	<0.001
**Radiologic findings (** ***n*** **= 216)**			
Cystic bronchiectasis in any lobes	83 (53.9)	39 (62.9)	0.291
Number of involved lobes	3 (2–5)	4 (3–5)	0.039
Modified Reiff score (*n* = 212)	6 (3–9)	8 (5–12)	0.030
**Comorbidities**			
Asthma, *n* (%)	42 (26.4)	23 (35.4)	0.238
Cardiovascular disease, *n* (%)	60 (37.7)	26 (40.0)	0.869
Diabetes mellitus, *n* (%) (*n* = 223)	20 (12.7)	4 (6.2)	0.235
Rhinosinusitis, *n* (%)	12 (7.5)	8 (12.3)	0.381
Neoplastic disease, *n* (%) (*n* = 223)	15 (9.5)	8 (12.3)	0.700
Tuberculosis, *n* (%) (*n* = 223)	71 (44.7)	25 (39.1)	0.540
**Pre-bronchodilator spirometry results (** ***n*** **= 216)**			
FVC (L)	2.5 (1.9–3.1)	2.2 (1.7–2.7)	0.006
FVC (%-predicted)	69.7 (59.0–79.2)	62.3 (50.3–75.8)	0.003
FEV_1_ (L)	1.4 (1.1–1.8)	1.1 (0.9–1.7)	0.002
FEV_1_ (%-predicted)	55.6 (44.8–65.4)	46.9 (35.1–59.6)	0.002
FEV_1_/FVC (%)	60.3 (51.9–65.7)	57.4 (47.2–65.5)	0.239
**Medication**			
Inhaler (*n* = 222)			
LABA or LAMA only, *n* (%)	19 (12.0)	4 (6.2)	0.300
ICS/LABA^†^, *n* (%)	13 (8.2)	7 (10.9)	0.704
LABA/LAMA^†^, *n* (%)	73 (46.2)	32 (50.0)	0.715
ICS/LABA/LAMA, *n* (%)	26 (16.5)	13 (20.3)	0.625
Oral drug (*n* = 223)			
Statin, *n* (%)	12 (7.5)	4 (6.2)	0.958
ACE inhibitor, *n* (%)	1 (0.6)	0 (0.0)	1.000
Proton pump inhibitor, *n* (%)	13 (8.2)	6 (9.4)	0.980

*Data are expressed as medians (interquartile ranges) for continuous variables and numbers (percentages) for categorical variables*.

* *Decreased QoL was defined as the lowest quartile of the total BHQ score (<57)*.

† *Six patients switched from one inhaler to the other*.

*COPD, chronic obstructive pulmonary disease; QoL, quality of life; BMI, body mass index; mMRC, modified Medical Research Council; FVC, forced vital capacity; FEV_1_, forced expiratory volume in 1 second; BSI, bronchiectasis severity index; PHQ-9, patient health questionnaire 9; FFS, fatigue severity scale; ICS, inhaled corticosteroid; LABA, long-acting β_2_ agonist; LAMA, long-acting muscarine antagonist; ACE, angiotensin-converting enzyme*.

### Factors Associated With Decreased QoL in Bronchiectasis With COPD

In univariable analyses, BMI [per each 1-kg/m^2^ decrease; unadjusted odds ratio (OR) = 1.12, 95% confidence interval (CI) = 1.03–1.23], mMRC ≥ 2 (unadjusted OR = 8.06, 95% CI = 4.13–16.25), PHQ-9 (per each 1-score increase, unadjusted OR = 1.40, 95% CI = 1.28–1.55), FFS (per each 1-score increase; unadjusted OR = 1.11, 95% CI = 1.08–1.14), FEV_1_ % predicted (per each 10% decrease; unadjusted OR = 1.33, 95% CI = 1.08–1.65), and modified Reiff score (per each 1-score increase; unadjusted OR = 1.08, 95% CI = 1.00–1.16) were significantly associated with decreased QoL. However, in multivariable analysis, mMRC ≥ 2 (adjusted OR = 3.21, 95% CI = 1.21–8.60), PHQ-9 (per each 1-score increase; adjusted OR = 1.28, 95% CI = 1.16–1.44), and FFS (per each 1-score increase; adjusted OR = 1.05, 95% CI = 1.01–1.09) were significantly associated with decreased QoL (Table 3).

**Table 3:** Factors associated with decreased quality of life in patients with coexisting COPD and bronchiectasis.

**Factors (*n* = 203)**	**Decreased quality of life (total BHQ score < 57)**			
	**Univariable analysis**		**Multivariable analysis**	
	**Unadjusted OR (95% CI)**	***p*-value**	**Adjusted OR (95% CI)**	***p*-value**
Age, per each 1-year increase	1.00 (0.97–1.03)	0.924	1.01 (0.97–1.06)	0.534
**Sex**				
Female	Ref.		Ref.	
Male	0.86 (0.46–1.57)	0.589	0.79 (0.21–2.87)	0.716
**Smoking history**				
Never-smoker	Ref.		Ref.	
Current or ex-smoker	0.88 (0.48–1.62)	0.690	0.90 (0.25–3.24)	0.866
BMI (kg/m^2^), per each 1-kg/m^2^ decrease	1.12 (1.03–1.23)	0.013	1.03 (0.91–1.17)	0.613
**Symptoms**				
mMRC <2	Ref.		Ref.	
mMRC ≥ 2	8.06 (4.13–16.25)	<0.001	3.21 (1.21–8.60)	0.019
PHQ-9, per each 1-score increase	1.40 (1.28–1.55)	<0.001	1.28 (1.16–1.44)	<0.001
FSS, per each 1-score increase	1.11 (1.08–1.14)	<0.001	1.05 (1.01–1.09)	0.020
**Acute exacerbation**				
No	Ref.		Ref.	
Yes	1.53 (0.82–2.95)	0.190	1.55 (0.58–4.34)	0.389
FEV_1_ % predicted, per each 10-percent decrease	1.33 (1.08–1.65)	0.008	0.94 (0.68–1.32)	0.730
Modified Reiff score, per each 1-score increase	1.08 (1.00–1.16)	0.046	1.06 (0.93–1.20)	0.373

*The ORs, CIs, and p-values were calculated from logistic regression analysis. In the multivariable model, all factors were included for adjustment*.

*COPD, chronic obstructive pulmonary disease; BHQ, bronchiectasis health questionnaire; BMI, body mass index; mMRC, modified Medical Research Council; PHQ-9, Patient Health Questionnaire 9; FSS, Fatigue Severity Score; FEV_1_, forced expiratory volume in 1 second; OR, odds ratio; CI, confidence interval*.

## Discussion

In this prospective observational study, we compared QoL scores in bronchiectasis patients in Korea according to COPD presence. We found that ~38% of patients with bronchiectasis have COPD, and these patients had lower QoL as measured by the BHQ than do patients without COPD. We additionally found that dyspnea estimated by mMRC (≥ 2), depression by PHQ-9, and fatigue by FSS were significant factors associated with decreased QoL among bronchiectasis patients with COPD.

The coexistence of COPD and bronchiectasis in Western countries is < 20% (1, 2, 4). Although COPD prevalence according to India's bronchiectasis registry was higher than the prevalences in Western countries, it was still only around 20% (3). In comparison, the COPD prevalence in the KMBARC registry was higher than expected, but it is not clear why. There are some possible explanations for the phenomenon. In this study, COPD was defined as physician-diagnosed COPD. Thus, the definition of COPD was not as strict as that in the previous Taiwan study that included smoking history as well as spirometric results (30). Another possibility is that that bronchiectasis with obstructive ventilatory impairment might have been regarded as having COPD. Bronchiectasis itself can cause obstructive ventilatory impairment (31). The high rate of COPD can be also attributable to the relatively high prevalence of asthma and TB in this cohort (14). The current Global Initiative for Chronic Obstructive Lung Disease (GOLD) recommendations indicate asthma or TB as a risk factor of COPD (32). We carefully suggest that asthmatic patients with fixed airflow obstruction and prior TB patients with airflow obstruction might have been regarded as COPD by attending physicians. Thus, the burden of COPD-related risk factors other than smoking was relatively high in our cohort, which might have led to a high rate of never-smokers in bronchiectasis patients with COPD. Although there are prevalence differences between our study and previous studies, these results suggest that at least one-fifth of bronchiectasis patients have COPD, and appropriate diagnosis and treatment are important for proper management.

It is well-recognized that bronchiectasis patients have lower QoL than patients without bronchiectasis (33), especially patients with exacerbations and respiratory symptoms (34). However, the QoL of bronchiectasis patients with COPD has rarely been evaluated. One European study analyzed bronchiectasis patients' QoL according to etiology, and patients with COPD-related bronchiectasis were shown to have lower QoL compared with patients with bronchiectasis caused by other etiologies (35). However, because that study evaluated QoL according to etiology, COPD patients whose COPD was not considered to be the cause of bronchiectasis were not included in the COPD group. By viewing COPD as comorbidity and not taking an etiology-based approach, our study showed that COPD significantly affected the QoL of patients with bronchiectasis. However, it should be mentioned that the minimal clinically important difference in the BHQ scores is not known. As a result, although the median difference of 1.7 units was statistically different in bronchiectasis patients by the presence or absence of COPD, this difference may not be clinically significant. Future studies are needed.

Among factors associated with QoL in bronchiectasis patients with COPD, dyspnea measured by mMRC had the most significant association with decreased QoL. This suggests that poorly controlled dyspnea can lead to poor QoL in bronchiectasis patients with COPD. Consistent with our results, a previous study using a COPD cohort showed that COPD patients with bronchiectasis have a high degree of dyspnea, which affected patients' QoL as measured by SGRQ score (12). Other important findings of our study are that depression and fatigue measured by PHQ-9 and FFS, respectively, affected QoL in bronchiectasis patients with COPD. Fatigue and depression are frequent in patients with COPD as well as those with bronchiectasis and have significant impacts on QoL in these patients (36–38). Accordingly, it can be postulated that these two conditions can affect the QoL in patients with bronchiectasis and COPD overlap. Regarding these conditions affecting the QoL in bronchiectasis patients with COPD, previous study findings suggest that these symptoms can be interactively connected, and one symptom may affect others. For example, a patient who complains of dyspnea may have unrecognized fatigue or depression as well. Thus, a strategy focusing on one component might not be as effective as a comprehensive approach to managing all these factors (e.g., bronchodilator use, pulmonary rehabilitation, as well as anti-depression treatment). Future studies are needed to optimize the assessment and treatment strategies to improve QoL in bronchiectasis patients with COPD.

There are some limitations to our study. First, this study was performed in Korean bronchiectasis patients. Thus, to generalize our findings, further studies using other bronchiectasis cohorts are needed. Second, because our study design was cross-sectional, we could not evaluate the association between COPD and longitudinal changes in QoL in bronchiectasis patients. Thus, future studies are needed. Third, although we suggested some potential reasons (TB, asthma, etc.) to explain the high proportion of never-smokers in bronchiectasis patients with COPD, the role of other important risk factors (e.g., biomass exposure) on this issue could not be evaluated due to the lack of data in KMBARC.

## Conclusions

COPD was a significant factor associated with decreased QoL in patients with bronchiectasis. Dyspnea, depression, and fatigue were associated with reduced QoL in bronchiectasis patients with COPD. In managing patients with bronchiectasis, appropriate diagnosis and treatment of COPD might help improve QoL.

## Data Availability Statement

The raw data supporting the conclusions of this article will be made available by the authors, without undue reservation.

## Ethics Statement

The study protocol was approved by the institutional review board of each institution that participated in the KMBARC, including Hallym University Chuncheon Sacred Heart Hospital (IRB number: 2018-07-006). All participants provided written informed consent.

## Author Contributions

HL and YK are guarantors of the manuscript. SK, HL, and YK designed the study and wrote the initial draft of the manuscript. SK performed data analysis. All authors were involved at all stages of the critical revision of the manuscript, read and approved the final manuscript, and meet the criteria for authorship as recommended by the International Committee of Medical Journal Editors.

## Conflict of Interest

The authors declare that the research was conducted in the absence of any commercial or financial relationships that could be construed as a potential conflict of interest.

## Publisher's Note

All claims expressed in this article are solely those of the authors and do not necessarily represent those of their affiliated organizations, or those of the publisher, the editors and the reviewers. Any product that may be evaluated in this article, or claim that may be made by its manufacturer, is not guaranteed or endorsed by the publisher.

## References

[1] Aksamit TR O'Donnell AE Barker A Olivier KN Winthrop KL Daniels MLA. Adult patients with bronchiectasis: a first look at the US bronchiectasis research registry. Chest. (2017) 151:982–92. 10.1016/j.chest.2016.10.055 27889361 PMC6026266

[2] Araújo D Shteinberg M Aliberti S Goeminne PC Hill AT Fardon TC. The independent contribution of Pseudomonas aeruginosa infection to long-term clinical outcomes in bronchiectasis. Eur Respir J. (2018) 51:1953. 10.1183/13993003.01953-2017 29386336

[3] Dhar R Singh S Talwar D Mohan M Tripathi SK Swarnakar R. Bronchiectasis in India: results from the European Multicentre Bronchiectasis Audit and Research Collaboration (EMBARC) and Respiratory Research Network of India Registry. Lancet Glob Health. (2019) 7:e1269–79. 10.1016/S2214-109X(19)30327-4 31402007

[4] Visser SK Bye PTP Fox GJ Burr LD Chang AB Holmes-Liew CL. Australian adults with bronchiectasis: the first report from the Australian Bronchiectasis Registry. Respir Med. (2019) 155:97–103. 10.1016/j.rmed.2019.07.016 31326739

[5] Choi H Yang B Nam H Kyoung DS Sim YS Park HY. Population-based prevalence of bronchiectasis and associated comorbidities in South Korea. Eur Respir J. (2019) 54:194. 10.1183/13993003.00194-2019 31048349

[6] Martinez-Garcia MA Miravitlles M. Bronchiectasis in COPD patients: more than a comorbidity? Int J Chron Obstruct Pulmon Dis. (2017) 12:1401. 10.2147/COPD.S132961 28546748 PMC5436792

[7] Ni Y Shi G Yu Y Hao J Chen T Song H. Clinical characteristics of patients with chronic obstructive pulmonary disease with comorbid bronchiectasis: a systemic review and meta-analysis. Int J Chron Obstruct Pulmon Dis. (2015) 10:1465–75. 10.2147/COPD.S83910 26251586 PMC4524532

[8] Gatheral T Kumar N Sansom B Lai D Nair A Vlahos I. COPD-related bronchiectasis; independent impact on disease course and outcomes. COPD. (2014) 11:605–14. 10.3109/15412555.2014.922174 24983298

[9] Patel IS Vlahos I Wilkinson TM Lloyd-Owen SJ Donaldson GC Wilks M. Bronchiectasis, exacerbation indices, and inflammation in chronic obstructive pulmonary disease. Am J Respir Crit Care Med. (2004) 170:400–7. 10.1164/rccm.200305-648OC 15130905

[10] Mao B Lu HW Li MH Fan LC Yang JW Miao XY. The existence of bronchiectasis predicts worse prognosis in patients with COPD. Sci Rep. (2015) 5:10961. 10.1038/srep10961 26077673 PMC4468518

[11] Ekici A Bulcun E Karakoc T Senturk E Ekici M. Factors associated with quality of life in subjects with stable COPD. Respir Care. (2015) 60:1585–91. 10.4187/respcare.03904 26152471

[12] Sahin H Naz I Susam S Erbaycu AE Olcay S. The effect of the presence and severity of bronchiectasis on the respiratory functions, exercise capacity, dyspnea perception, and quality of life in patients with chronic obstructive pulmonary disease. Ann Thorac Med. (2020) 15:26–32. 10.4103/atm.ATM_198_19 32002044 PMC6967141

[13] Lee H Choi H Sim YS Park S Kim WJ Yoo KH. KMBARC registry: protocol for a multicentre observational cohort study on non-cystic fibrosis bronchiectasis in Korea. BMJ Open. (2020) 10:e034090. 10.1136/bmjopen-2019-034090 31959610 PMC7044940

[14] Lee H Choi H Chalmers JD Dhar R Nguyen TQ Visser SK. Characteristics of bronchiectasis in Korea: first data from the Korean Multicentre Bronchiectasis Audit and Research Collaboration registry and comparison with other international registries. Respirology. (2021) 26:619–21. 10.1111/resp.14059 33876470

[15] Kim HK Lee H Kim S-H Choi H Lee JH Lee JS. Validation of the Korean version of the bronchiectasis health questionnaire. Tuberc Respir Dis. (2020) 83:228–33. 10.4046/trd.2020.0025 32578411 PMC7362752

[16] Hill AT Sullivan AL Chalmers JD De Soyza A Elborn JS Floto RA. British Thoracic Society Guideline for bronchiectasis in adults. Thorax. (2019) 74:1–69. 10.1136/thoraxjnl-2018-212463 30545985

[17] Spinou A Siegert RJ Guan WJ Patel AS Gosker HR Lee KK. The development and validation of the bronchiectasis health questionnaire. Eur Respir J. (2017) 49:1532. 10.1183/13993003.01532-2016 28495688

[18] Mahler DA Wells CK. Evaluation of clinical methods for rating dyspnea. Chest. (1988) 93:580–6. 10.1378/chest.93.3.580 3342669

[19] Murray M Pentland J Turnbull K MacQuarrie S Hill A. Sputum colour: a useful clinical tool in non-cystic fibrosis bronchiectasis. Eur Respiratory J. (2009) 34:361–4. 10.1183/09031936.00163208 19648517

[20] Hill AT Haworth CS Aliberti S Barker A Blasi F Boersma W. Pulmonary exacerbation in adults with bronchiectasis: a consensus definition for clinical research. Eur Respir J. (2017) 49:51. 10.1183/13993003.00051-2017 28596426

[21] Chalmers JD Goeminne P Aliberti S McDonnell MJ Lonni S Davidson J. The bronchiectasis severity index. An international derivation and validation study. Am J Respir Crit Care Med. (2014) 189:576–85. 10.1164/rccm.201309-1575OC 24328736 PMC3977711

[22] Martínez-García M de Gracia J Vendrell Relat M Girón RM Máiz Carro L de la Rosa Carrillo D. Multidimensional approach to non-cystic fibrosis bronchiectasis: the FACED score. Eur Respir J. (2014) 43:1357–67. 10.1183/09031936.00026313 24232697

[23] Kroenke K Spitzer RL Williams JB. The PHQ-9: validity of a brief depression severity measure. J Gen Intern Med. (2001) 16:606–13. 10.1046/j.1525-1497.2001.016009606.x 11556941 PMC1495268

[24] Park S-J Choi H-R Choi J-H Kim K-W Hong J-P. Reliability and validity of the Korean version of the Patient Health Questionnaire-9 (PHQ-9). Anxiety Mood. (2010) 6:119–24.

[25] Krupp LB LaRocca NG Muir-Nash J Steinberg AD. The fatigue severity scale. Application to patients with multiple sclerosis and systemic lupus erythematosus. Arch Neurol. (1989) 46:1121–3. 10.1001/archneur.1989.00520460115022 2803071

[26] Lee JH Jeong HS Lim SM Cho HB Ma J-Y Ko E. Reliability and validity of the fatigue severity scale among University Student in South Korea. Korean J Biol Psychiatry. (2013) 20:6–11. Available online at: http://journal.biolpsychiatry.or.kr/asp/journal_fulltxt.asp?no=0092013002&m=0 (accessed July 29, 2021).

[27] Mandal P Patel D Hill A. A simplified CT scoring system in non-cystic fibrosis bronchiectasis. Eur Respiratory Soc. (2013) 42:P241. Available online at: https://erj.ersjournals.com/content/42/Suppl_57/P241.short (accessed July 29, 2021).

[28] Miller MR Hankinson J Brusasco V Burgos F Casaburi R Coates A. Standardisation of spirometry. Eur Respir J. (2005) 26:319–38. 10.1183/09031936.05.00034805 16055882

[29] Choi JK Paek D Lee JO. Normal predictive values of spirometry in Korean population. Tuberc Respir Dis. (2005) 58:230–42. 10.4046/trd.2005.58.3.230

[30] Huang H-Y Chung F-T Lo C-Y Lin H-C Huang Y-T Yeh C-H. Etiology and characteristics of patients with bronchiectasis in Taiwan: a cohort study from 2002 to 2016. BMC Pulm Med. (2020) 20:45. 10.1186/s12890-020-1080-7 32070324 PMC7029505

[31] Yang B Jang HJ Chung SJ Yoo SJ Kim T Kim SH. Factors associated with bronchiectasis in Korea: a national database study. Ann Transl Med. (2020) 8:1350. 10.21037/atm-20-4873 33313095 PMC7723591

[32] GOLD. Global Strategy for the Diagnosis, Management and Prevention of Chronic Obstructive Pulmonary Disease. (2021). Available online at: http://www.goldcopd.org (accessed July 29, 2021).

[33] Yang B Choi H Lim JH Park HY Kang D Cho J. The disease burden of bronchiectasis in comparison with chronic obstructive pulmonary disease: a national database study in Korea. Ann Trans Med. (2019) 7:770. 10.21037/atm.2019.11.55 32042786 PMC6989976

[34] Gao YH Abo Leyah H Finch S Lonergan M Aliberti S De Soyza A. Relationship between symptoms, exacerbations, and treatment response in bronchiectasis. Am J Respir Crit Care Med. (2020) 201:1499–507. 10.1164/rccm.201910-1972OC 32097051

[35] Terpstra LC Biesenbeek S Altenburg J Boersma WG. Aetiology and disease severity are among the determinants of quality of life in bronchiectasis. Clin Respir J. (2019) 13:521–9. 10.1111/crj.13054 31295770

[36] Kouijzer M Brusse-Keizer M Bode C. COPD-related fatigue: impact on daily life and treatment opportunities from the patient's perspective. Respir Med. (2018) 141:47–51. 10.1016/j.rmed.2018.06.011 30053971

[37] Lee H Jhun BW Cho J Yoo KH Lee JH Kim DK. Different impacts of respiratory symptoms and comorbidities on COPD-specific health-related quality of life by COPD severity. Int J Chron Obstruct Pulmon Dis. (2017) 12:3301–10. 10.2147/COPD.S145910 29180860 PMC5691931

[38] Olveira C Olveira G Gaspar I Dorado A Cruz I Soriguer F. Depression and anxiety symptoms in bronchiectasis: associations with health-related quality of life. Qual Life Res. (2013) 22:597–605. 10.1007/s11136-012-0188-5 22544417

